# Antidiabetic and Antioxidant Properties of *Triticum aestivum* in Streptozotocin-Induced Diabetic Rats

**DOI:** 10.1155/2013/716073

**Published:** 2013-12-14

**Authors:** Yogesha Mohan, Grace Nirmala Jesuthankaraj, Narendhirakannan Ramasamy Thangavelu

**Affiliations:** Department of Biotechnology, School of Biotechnology and Health Sciences, Karunya Institute of Technology and Sciences, Karunya University, Karunya Nagar, Coimbatore, Tamil Nadu 641 114, India

## Abstract

The antidiabetic and antioxidant potential of *Triticum aestivum* were evaluated by using *in vivo* methods in normal and streptozotocin-induced diabetic rats. Diabetes was induced in the Wistar strain albino rats by injecting streptozotocin at a dose of 55 mg/kg body weight. Ethanolic extracts of *Triticum aestivum* at doses of 100 mg/kg body weight were administered orally for 30 days. Various parameters were studied and the treatment group with the extract showed a significant increase in the liver glycogen and a significant decrease in fasting blood glucose, glycosylated hemoglobin levels, and serum marker enzyme levels. The total cholesterol and serum triglycerides levels, low density lipoprotein, and very low density lipoprotein were also significantly reduced and the high density lipoprotein level was significantly increased upon treatment with the *Triticum aestivum* ethanol extract. A significant decrease in the levels of lipid peroxides, superoxide dismutase, and glutathione peroxidise and increase in the levels of vitamin E, catalase, and reduced glutathione were observed in *Triticum aestivum* treated diabetic rats. Thus, from this study we conclude that ethanolic extract of *Triticum aestivum* exhibited significant antihyperglycemic, hypolipidemic, and antioxidant activities in streptozotocin-induced diabetic rats.

## 1. Introduction

Diabetes mellitus (DM) is a chronic endocrine disorder, involving metabolic disorders of carbohydrate, fat, and protein. All forms of diabetes are characterized by a decrease in the circulating concentration of insulin (insulin deficiency) and a decrease in the response of peripheral tissues to insulin (insulin resistance) [1]. The disorder has reached epidemic levels and threatens a worldwide epidemic. According to World Health Organization (WHO), the disease incidence in 2010 was about 285 million people worldwide, and the number is projected to grow to 438 million by 2030 [2]. India has earned the dubious distinction of being termed the “Diabetes Capital of the World” by leading the world with largest number of diabetic subjects and the prevalence of diabetes is consistently increasing [3].

Oxidative stress is involved in the development and progression of diabetes-associated complications. In hyperglycemic condition, continuous generation of reactive oxygen species (ROS) occurs and the evidence showed diabetes induced changes in the activities of antioxidant enzymes in various tissues. Antioxidants play an important role in scavenging the free radicals and protect the human body from oxidative stress [4–6]. Hence, drug with both antioxidant and antidiabetic property would be useful for the treatment of the diabetes mellitus. In recent times, many medicinal plants have been reported to cure diabetes worldwide and have been used widely as antidiabetic remedies. WHO has suggested the evaluation of traditional plant treatments for diabetes as they are effective, nontoxic, with less or no side effects, and are considered to be excellent candidates for oral therapy [7]. The antihyperglycemic activity of the plants is mainly due to their ability to restore the function of pancreatic tissues by causing an increase in insulin output or inhibiting the intestinal absorption of glucose or to the facilitation of metabolites in insulin-dependent processes. Glycosides, alkaloids, terpenoids, flavonoids, carotenoids, and so forth, from the plants, are frequently implicated in having antidiabetic effect [8].

Wheatgrass is the young grass of common wheat plant, *Triticum aestivum *Linn., belonging to the family Poaceae (Gramineae). It is commonly known for its high chlorophyll content which accounts for 70% of its chemical constituents. Wheatgrass has been used as traditional herbal medicine and is highly valued for its therapeutic and nutritional properties [9]. The use of wheatgrass juice for therapeutic purposes was developed and popularized by Dr. Wigmore (1909–1996), as a part of her therapeutic nutritional approach [10]. The therapeutic qualities of *Triticum aestivum* have been attributed to its rich nutritional content, including chlorophyll, vitamins (A, C, and E), bioflavonoids, iron, minerals (calcium and magnesium), and 17 amino acids, 8 of which are essential [11]. Very little clinical data exists to support the use of *Triticum aestivum*, even though it was recommended for four decades as a treatment for various illnesses.

*Triticum aestivum* was found to reduce symptoms in patients with rheumatoid arthritis [12], to reduce severity of rectal bleeding in patients with ulcerative colitis [13], and to reduce the frequency of blood transfusions in patients with thalassemia major [14]. Therefore, the present study was carried out to evaluate the antioxidant, antihyperlipidemic, and antidiabetic potential of *Triticum aestivum* in streptozotocin-induced diabetic rats.

## 2. Materials and Methods

### 2.1. Growing of Wheatgrass

The grass of *T. aestivum *used in this study was grown under indoor conditions. Overnight soaked *T. aestivum *seeds were used to cultivate. Little quantities of water were sprinkled evenly over soil and 3-4 hours of indirect sunlight projection was allowed daily for growth of grass. On the seventh day, grass is harvested and used for further studies.

### 2.2. Preparation of Plant Extract

The harvested wheatgrass (*T. aestivum*) is washed, then dried at room temperature. The dried grass was subjected to size reduction to a coarse powder by using dry grinder and passed through sieve. This powder was packed into soxhlet apparatus and subjected to hot continuous percolation using ethanol. The extract was then filtered and concentrated under reduced pressure using a rotator evaporator at 40°C until the solvent completely dried. The yield of the ethanolic extract was 30%. The extract obtained was then dissolved in 2% of gum *Acacia* for the pharmacological studies.

### 2.3. Preliminary Phytochemical Screening

The ethanol extract of *T. aestivum *was screened for the presence of various phytoconstituents like steroids, alkaloids, glycosides, flavonoids, carbohydrates, amino acids, saponins, terpenoids, tannins, and phenolic compounds [15–17].

### 2.4. Antidiabetic Activity

#### 2.4.1. Animals

Male albino Wistar rats (150–200 g body weight) were obtained from College of Veterinary and Animal Sciences, Thrissur, India, and were maintained under a constant 12-hour light and dark cycle at 21–23°C. The animals were maintained in accordance with the guidelines of the National Institute of Nutrition, Hyderabad, India. The study was approved by the Institutional Ethics Committee. Throughout the experimental period, all four groups of animals were fed with a normal laboratory chow standard pellet diet (Sai feeds, Banglore, India) and water* ad libitum*.

#### 2.4.2. Experimental Induction of Diabetes

Animals were allowed to fast for 12 h and were administered freshly prepared streptozotocin (STZ) (Himedia) at the concentration of 55 mg/kg bodyweight, i.p. in 0.1 mol/L cold citrate buffer, pH 4.5 [18]. The STZ-treated animals were allowed to drink 5% glucose solution overnight to overcome drug-induced hypoglycemia. Rats having persistent glycosuria and hyperglycaemia with a fasting blood glucose >250 mg/dL on the third day after the STZ injection were considered diabetic and were used for further experimentation.

#### 2.4.3. Experimental Design

Animals were divided into four groups, consisting of a minimum of six animals each, as follows:

 Group I, control rats receiving 0.1 mol/L citrate buffer (pH 4.5);
 Group II, diabetic control;
 Group III, diabetic rats were administered 100 mg/kg wheatgrass (*T. aestivum*) ethanolic extract per day orally for 30 days;
 Group IV, diabetic rats were administered 10 mg/kg glibenclamide solution orally [19] per day for 30 days.

One week after the induction of diabetes in Wistar rats, the fasting blood glucose levels of fasted rats were measured. Rats with blood glucose >250 mg/dL were included in the study. They were divided into four groups with six rats in each group. Doses of (100 mg/kg body weight) of the plant extracts were given every day till the completion of the experiment (i.e., 30 days), whereas untreated and diabetic control groups were given 0.1 mol/L citrate buffer every day orally.

At the end of the experiment, the blood was collected for biochemical studies. The serum was then separated by centrifugation and was either assayed immediately or stored at −20°C.

#### 2.4.4. Biochemical Estimations

Blood was collected from the tail vein of the overnight fasting rat at 0th (before the start of the experiment), 3rd day, 10th day, 17th day, 24th day, and 30th day and the glucose levels were estimated by using Accu-Check Active glucometer. Weight of individual animals was measured gravimetrically on 0th and 30th days of the experiment. Urine glucose assessment is done by using Diastrips on 0th, 3rd day, 10th day, 17thday, 24th day and 30th day. The lipid profiles (total cholesterol, TG, HDL, and LDL) for all the four groups of animals were performed using commercially available kits as described earlier [20]. Glycogen content of liver was measured according to Van method [21].

Hexokinase was assayed according to the method of Brandstrup et al., [22]. Lactate dehydrogenase was assayed according to the method of King [23]. Glucose-6-phosphatase was assayed according to the method of Hikaru and Toshitsugu [24]. The glycosylated hemoglobin *in vitro *was measured colorimetrically, as suggested by Nayak and Pattabiraman [25]. These assays were done at the end of the 30th day of the experiments.

### 2.5. *In Vivo *Antioxidant Activity

The activities of all the antioxidant enzymes were estimated in liver tissue homogenates. Reduced glutathione (GSH) was estimated by the method of Ellman [26]. SOD was assayed by the method of Misra and Fridovich [27]. GPx was assayed by the method of Rotruck et al. [28]. CAT was assayed by the method of Takahara et al. [29]. Vitamin E was estimated by the method of Desai [30]. Lipid peroxides were estimated in liver tissue homogenates using thiobarbituric acid reactive substances (TBARS) by the method of Ohkawa et al. [31].

### 2.6. Statistical Analysis

Results were expressed as the mean ± standard deviation. Data was statistically analyzed using one-way ANOVA as primary test followed by Dunnett's test using GraphPad InStat 3.0 software for Windows XP and GraphPad software, San Diego, CA, USA.

## 3. Results

Table 1 shows the phytochemical constituents of *Triticum aestivum* extract. The preliminary phytochemical studies indicated the presence of alkaloids, saponins, steroids, flavonoids, tannins, and glycosides.  Table 2 represents the changes in body weight in normal and experimental diabetic rats. STZ produced significant loss in body weight as compared to normal rats during the study. Diabetic control rats continued to lose weight till the end of the study while *Triticum aestivum* treated rats at a dose (100 mg/kg body weight) showed significant improvement in body weight compared to diabetic control group. There was no significant difference between the *Triticum aestivum* and glibenclamide-treated groups. Results of the effect of *Triticum aestivum* on blood glucose level of STZ-induced diabetic rats are presented in Table 3. From the first week onwards, a significant antihyperglycemic effect was marked. The reduction in blood glucose was maximum on the fourth week, receiving 100 mg/kg body weight of *Triticum aestivum* and the results were comparable with that of glibenclamide. STZ caused a significant increase in blood glucose level. The results of the present study clearly indicated that the ethanolic extract exhibited significant antidiabetic activity in STZ-diabetic rats, comparable to the effect exhibited by standard drug glibenclamide.

After 30 days treatment period, it was observed that the animals treated with *T. aestivum* extract (100 mg/kg) and glibenclamide showed significant decrease in diabetes induced urine glucose level (Table 4).  Table 5 shows the level of glycosylated hemoglobin in normal and experimental rats. A significant (*P* < 0.05) increase in the level of glycosylated hemoglobin was observed in diabetic rats when compared to control rats (group 1). Administration of *Triticum aestivum* (group 3) and glibenclamide (group 4) to diabetic rats significantly (*P* < 0.05) reverted glucose levels to near-normal level. The mobilization of glucose into liver and skeletal muscle was significantly decreased (*P* < 0.001) with experimentally induced diabetes mellitus. There was a significant increase in glycogen content of liver (*P* < 0.001) with *Triticum aestivum* or glibenclamide treatment as compared to diabetic control.

**Figure 1:**
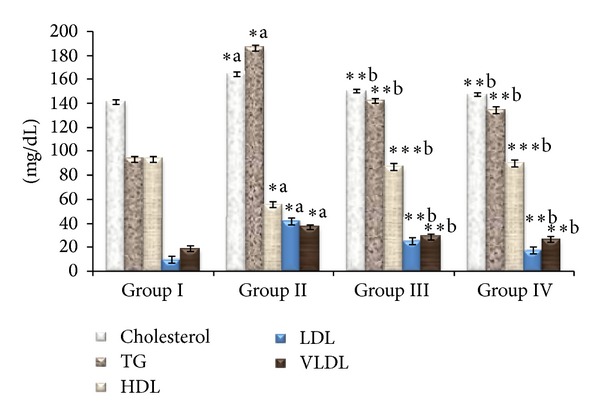
Effect of ethanolic extract of *Triticum aestivum *on the total cholesterol (TC), triglyceride (TG), HDL, LDL, and VLDL levels in STZ-induced rats. Values are the mean ± SD for six animals in each group. Values are statistically significant at *P < 0.05, **P < 0.001, and ***P < 0.01; statistical significance was compared within the groups as follows. ^a^Diabetic rats were compared with normal rats. ^b^Glibenclamide and *Triticum aestivum *treated diabetic rats were compared with diabetic rats.

Figure 1 shows the level of plasma lipid profile such as triglyceride (TG), total cholesterol (TC), high-density lipoprotein (HDL), low-density lipoprotein (LDL), and very low-density lipoprotein (VLDL). It was observed that due to diabetes there was an increase in the total cholesterol levels as well as triglyceride levels. Plasma TG, TC, LDL, and VLDL levels were significantly elevated and HDL level was decreased in diabetic rats when compared with control rats. Treatment with *Triticum aestivum* reversed the diabetes-induced hyperlipidemia. A significant reduction of TG, TC, LDL, and VLDL levels (*P* < 0.001) and increase in HDL level were observed after the treatment with *Triticum aestivum *(*P* < 0.01).

**Figure 2:**
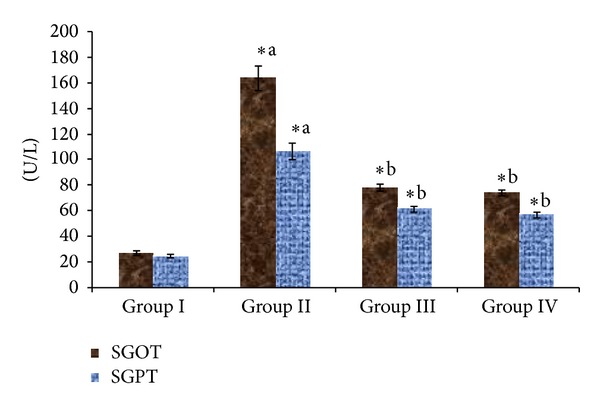
Effect of ethanolic extract of *Triticum aestivum *on the SGOT and SGPT levels in STZ-induced model. Values are the mean ± SD for six animals in each group. Values are statistically significant at *P < 0.05; statistical significance was compared within the groups as follows. ^a^Diabetic rats were compared with normal rats. ^b^Glibenclamide and *Triticum aestivum *treated diabetic rats were compared with diabetic rats.

Figure 2 showed the effect of *Triticum aestivum* extract administration on serum markers enzymes. The concentration of SGOT and SGPT was increased in diabetes condition when compared with normal control. Administration of *Triticum aestivum *ethanol extract and glibenclamide reduced these levels compared to diabetic rats.

**Figure 3:**
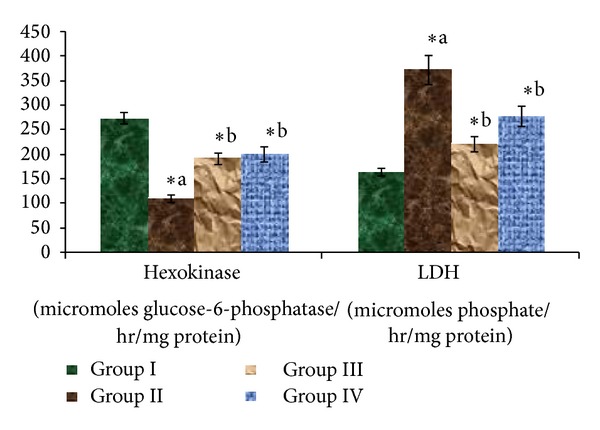
Effect of ethanolic extract of *Triticum aestivum *on the hexokinase and LDH levels in STZ-induced model. Values are the mean ± SD for six animals in each group. Values are statistically significant at *P < 0.05; statistical significance was compared within the groups as follows. ^a^Diabetic rats were compared with normal rats. ^b^Glibenclamide and *Triticum aestivum *treated diabetic rats were compared with diabetic rats.

**Figure 4:**
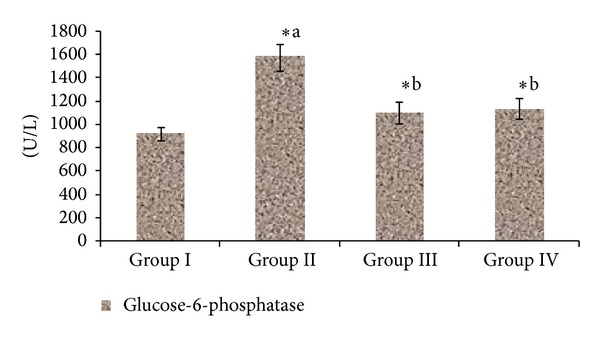
Effect of ethanolic extract of *Triticum aestivum *on the glucose-6-phosphatase level in STZ-induced model. Values are the mean ± SD for six animals in each group. Values are statistically significant at *P < 0.05; statistical significance was compared within the groups as follows. ^a^Diabetic rats were compared with normal rats. ^b^Glibenclamide and *Triticum aestivum *treated diabetic rats were compared with diabetic rats.

Figure 3 represents the activity of hexokinase and lactate dehydrogenase levels. The hexokinase enzyme activity was suppressed in STZ-induced diabetic rats while lactate dehydrogenase levels increased in STZ-induced diabetic rats when compared to normal rats. This activity was restored by both *Triticum aestivum* and glibenclamide treatment in experimental diabetic rats. However, 4 weeks of oral administration of *Triticum aestivum* at the doses of 100 mg/kg body weight and glibenclamide tended to bring these values back to normal.  Figure 4 represents the activity of glucose-6-phosphatase levels. The activity of hepatic hexokinase decreased, whereas the activities of glucose-6-phosphatase increased in STZ-induced diabetic rats which were reverted to near normal by the administration of *Triticum aestivum *and glibenclamide.

**Figure 5:**
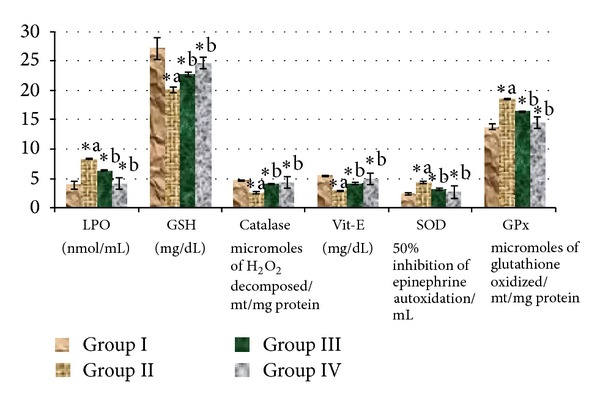
Effect of ethanolic extract of *Triticum aestivum *on the antioxidant levels of liver homogenates in STZ-induced model. Values are the mean ± SD for six animals in each group. Values are statistically significant at *P < 0.05; statistical significance was compared within the groups as follows. ^a^Diabetic rats were compared with normal rats. ^b^Glibenclamide and *Triticum aestivum *treated diabetic rats were compared with diabetic rats.

There was a significant (*P* < 0.05) elevation in TBARS, SOD, and GPx levels and reduction in GSH, CAT, and vitamin E levels in liver of diabetic rats compared to control rats. The administration of *Triticum aestivum* 100 mg/kg and glibenclamide significantly (*P* < 0.05) reversed these changes to near-normal level (Figure 5). A significant greater efficacy was observed in *Triticum aestivum* treated diabetic rats when compared with diabetic rats.

## 4. Discussion

The present study discusses the antioxidant and antidiabetic effects of the ethanolic extract of *Triticum aestivum* on streptozotocin-induced diabetic rats. Insulin-dependent diabetes mellitus (IDDM) is a disease caused by progressive destruction of the insulin secreting *β*-cells. Streptozotocin (STZ) is most commonly used to induce diabetes in rats. This causes the death of pancreatic *β*-cell by alkylation of DNA resulting in reduced synthesis and release of insulin. This results in fragmentation of DNA by means of production of reactive oxygen species [32, 33]. STZ selectively destroys the pancreatic cells that secrete insulin, which causes less active pancreatic cells and produces diabetes mellitus [34]. There should be many surviving *β*-cells even after treatment with a low dose of STZ and regeneration is also possible [35]. From the results of the present study, it appears that insulin-producing cells are still functioning and the stimulation of insulin release could be responsible for most of the metabolic effects. The medicinal plant compounds may have mechanisms acting as insulin-like effect, improving insulin sensitivity, augmenting glucose-dependent insulin secretion, and stimulating the regeneration of islets of Langerhans in pancreas of STZ-induced diabetic rats. At present, the only option to achieve permanent normoglycemia in diabetic patients is renewal of the *β*-cells or stimulating insulin secretion by medicinal plant [36].

Presence of phenolic compounds such as alkaloids, flavonoids, and tannins in *Triticum aestivum* was noticed in preliminary phytochemical analysis. We hypothesized that *Triticum aestivum *can be attributed to their insulin-trophic effect that enables the reduction of blood glucose levels due to their antidiabetic effect. The possible mechanism of action of *Triticum aestivum* extract treated groups could be potentiating the pancreatic secretion of insulin from *β*-cells of islets, as was evident by significantly lowering the level of glucose. Induction of diabetes by STZ causes loss of body weight due to the increased muscle wasting and loss of tissue proteins [37]. Due to destruction of *β*-cells, the decrease in body weight and increase in food and water intake were commonly observed in diabetes and this may be due to metabolic changes caused by lack or deficiency of insulin [38].

In diabetic rats, reduction in body weight changes observed might be the result of degradation of structural proteins due to deficiency of carbohydrate for the energy metabolism [39]. A significant increase in body weight of diabetic rats treated with wheatgrass showed the blood glucose stabilization effect which in turn prevents the loss of body weight. Administration of wheatgrass lowers blood glucose in STZ-induced diabetic rats significantly and its effect was almost equal to that of glibenclamide. The reference drug glibenclamide is an ATP-sensitive K + channel blocker used to treat noninsulin-dependent diabetes. It appears to be a useful addition to the range of oral hypoglycemic sulphonylureas, but because of its potency (and its expense) it is probably best used as an antidiabetic drug [40–42]. Diabetic rats treated with the wheatgrass extract showed an improvement in body weight in comparison to the diabetic control and standard glibenclamide-treated groups, which signifies the reversal of gluconeogenesis. Further, this antidiabetic activity indicates that wheatgrass may stimulate insulin secretion from the remaining *β*-cells or regenerated *β*-cells.

Urine glucose estimation study indicated that the animals treated with ethanolic extract showed significant decrease in diabetes induced urine glucose level when compared with the normal level. A marked reduction in blood glucose level and urine glucose level toward normal level suggested antidiabetic potential of the plant.

In diabetic rats, the levels of HbA1C are increased due to the persistent hyperglycemia which results in glycation of haemoglobin. The concentration of HbA1C is related to diabetic retinopathy, nephropathy, and neuropathy and it is considered as a tool for the diagnosis and prognosis of diabetes-associated complications [43]. The synthesis of haemoglobin is reduced in diabetic rats [44]. In our study, administration of wheatgrass significantly decreased the HbA1C and increased Hb levels in diabetic rats. The ability of wheatgrass to decrease HbA1C levels in diabetic rats showed its potentials to prevent the diabetic-associated complications.

Glycogen levels in liver were low in diabetic animals but in *Triticum aestivum* treated diabetic animals it increased several folds (Table 5). Glycogen levels in diabetic rats were found to be very low despite high blood glucose levels possibly due to lower levels of glycogen synthase activity. Accumulation of glycogen in liver of treated animals is somewhat similar to that reported during insulin therapy [45–48].

In the present study, the significant increase in serum SGOT and SGPT levels that was observed in STZ-induced diabetic rats represents liver damage compared to control rats. Liver necrosis in STZ-induced diabetic rats increased the activities of SGPT and SGOT in plasma by leakage of these enzymes from liver cytosol into the blood stream. Oral administration of wheatgrass showed its protective nature on liver tissue by reducing the elevated levels of SGOT and SGPT. In normal rats, insulin activates the lipolytic hormones action on the peripheral fat which hydrolyses triglycerides and prevents mobilization of free fatty acids [49]. Nevertheless, insulin deficiency inactivates the lipoprotein lipase promoting liver conversion of free fatty acids into phospholipids and cholesterol which get discharged into blood resulting in elevated serum phospholipid level [50]. The elevated cholesterol, triglycerides, and LDL levels and decreased HDL levels were reported in diabetic patients [51]. In this study, administration of wheatgrass significantly reduced elevated total cholesterol, triglycerides, VLDL, and LDL levels in diabetic rats. Also, increased level of HDL was observed in diabetic rats treated with both the doses of wheatgrass and glibenclamide compared to diabetic control rats. This action of wheatgrass renders its lipid lowering activity in diabetic condition and hence prevents diabetic-associated complications.

Persistent hyperglycemia is a major contributor to metabolic alterations that lead to the pathogenesis of diabetic complications. One of the key enzymes in the catabolism of glucose is hexokinase, which phosphorylates glucose and converts it into glucose-6-phosphatase [52]. The activity of this enzyme got decreased in the liver of streptozotocin diabetic rats. Administration of wheatgrass to diabetic rats resulted in an increased activity of liver hexokinase. The increased activity of hexokinase helps in increasing glycolysis and increasing utilization of glucose for energy production. wheatgrass has been observed to reduce the levels of glucose in the blood [53]. The decrease in the concentration of blood glucose in wheatgrass-treated rats also can be due to increased glycolysis (increased liver hexokinase activity). The activities of hepatic glucose-6-phosphatase were increased significantly in diabetic rats [54], which is the regulatory enzyme in gluconeogenic pathway. Lactate dehydrogenase catalyses the conversion of pyruvate to lactate, which is subsequently converted to glucose in gluconeogenic flux and it is a functional enzyme in anaerobic glycolysis. Increased LDH activity in diabetes mellitus has been reported by Pozzilli et al. [55]. Diabetic rats treated with wheatgrass had significantly restored LDH activity, probably as a result of the stimulation of the oxidation of NADH. Normal LDH activity is indicative of improved channelling of (pyruvate) glucose for mitochondrial oxidation.

Diabetes is marked by increased production of free radicals or impaired antioxidant defenses. The generation of superoxide anion radicals by glucose oxidization and its dismutation to hydrogen peroxide leads to the formation of reactive hydroxyl radicals [56, 57]. In present study, elevated TBARS, SOD, and GPx levels and reduced GSH, CAT, vitamin E levels were observed in STZ-induced diabetic rats compared to control rats. These changes may be due to the glucose oxidation, formation of free radical generation, and nitric oxide donor property of STZ [58]. Administration of wheatgrass significantly reduced TBARS, SOD, and GPx levels and increased CAT, vitamin E, and GSH levels in diabetic rats. The action of the wheatgrass to restore the altered antioxidant enzymes in STZ-induced diabetic rats indicates its free radical scavenging potential.

The ethanolic extracts have been chosen because of its expected flavonoid contents that were reported to have antidiabetic activity. The activities of phenolics having antioxidant activity further confirms this view [59]. The phytochemical analysis of wheatgrass showed the presence of tannins, flavonoids, saponins, and sterols. Their antidiabetic ability to regenerate the pancreatic *β*-cell has already been proved [60]. Sterols can decrease blood sugar in experimental animal models [61]. The antioxidant activity of the phenolic, tannins, and flavonoid compounds are attributed to its redox properties which can act as reducing agents, hydrogen donators, and singlet oxygen quenchers [62]. Polyphenolics having hydroxyl groups are very important plant constituents which can protect body from oxidative stress [63]. Flavones glycosides have also been reported as strong antioxidants and potent hypoglycemic agents. The present study clearly concluded that ethanol extract of wheatgrass possesses the ability to control blood glucose in diabetes. Its antihyperglycemic and free radical scavenging property has potential to prevent diabetic-associated complications. Our current investigation supports the traditional use of wheatgrass in the treatment of diabetes. This study is limited by its animal model design and further investigations are to be done to infer clinical correlations to humans.

## Figures and Tables

**Table 1:** Phytochemical constituents of *Triticum aestivum* extract.

Phytochemicals	Presence/absence
Alkaloids	Present
Flavonoids	Present
Glycosides	Present
Saponins	Present
Tannins	Present
Phytosterols	Present
Triterpenoid	Present
Amino acids	Present
Protein	Present

**Table 2:** Effect of *Triticum aestivum* on body weight in streptozotocin-induced diabetic rats.

Treatment	Body weight (g)	
	Day 1	Day 30
Normal control	150 ± 6.9	152 ± 6.5
Diabetic control	151 ± 7.1	110 ± 3.2
Diabetic + treated	151.5 ± 6.5	128 ± 5.9*

Diabetic + glibenclamide	152.1 ± 5.3	137 ± 5.6*

Values are expressed as mean ± SD (*n* = 6 rats). *Significance level among different groups at *P* < 0.05.

**Table 3:** Effect of *Triticum aestivum* on the blood glucose level in streptozotocin-induced diabetic rats.

Treatment	Fasting blood glucose concentration (mg/dL)					
	Day 0	Day 3	Day 7	Day 14	Day 21	Day 30
Normal control	94.6 ± 2.5	94 ± 1.5	95 ± 2.1	95.9 ± 1.9	96 ± 4.7	96 ± 1.7
Diabetic control	97.2 ± 4.3	309 ± 5.3	365 ± 7.4	412 ± 6.5	418 ± 4.3	422 ± 5.4
Diabetic + treated	97 ± 2.8	305 ± 4.4	238 ± 3.7	175 ± 4.9	135 ± 8.8	113 ± 3.5*
Diabetic + glibenclamide	96.5 ± 3.1	303 ± 2.9	215 ± 4.9	161 ± 3.3	125 ± 6.7	106 ± 2.5*

Values are expressed as mean ± SD (*n* = 6 rats). *Significance level among different groups at *P* < 0.05.

**Table 4:** Effect of *Triticum aestivum* on the urine glucose level in streptozotocin-induced diabetic rats.

Treatment	Intensity of glucose in urine					
	Day 0	Day 3	Day 7	Day 14	Day 21	Day 30
Normal control	Nil	Nil	Nil	Nil	Nil	Nil
Diabetic control	Nil	+++	++++	++++	++++	++++
Diabetic + treated	Nil	+++	++++	+++	++	+
Diabetic + glibenclamide	Nil	+++	++++	++	+	+

Intensity of glucose in urine, +: mild, ++: moderate, +++: higher, ++++: severe.

**Table 5:** Effect of *Triticum aestivum* on the HbA1C and liver glycogen level in streptozotocin-induced diabetic rats.

Treatment	HbA1C	Liver glycogen (mg/g)
Normal control	5.4%	16.53 ± 0.16
Diabetic control	8.7%	8.12 ± 0.12*
Diabetic + treated	7.8%	12.98 ± 1.1*
Diabetic + glibenclamide	7.5%	14.58 ± 0.43*

Values are expressed as mean ± SD (*n* = 6 rats). *Significance level among different groups at *P* < 0.001.
